# Transplantation of Adipose Stromal Cell Sheet Producing Hepatocyte Growth Factor Induces Pleiotropic Effect in Ischemic Skeletal Muscle

**DOI:** 10.3390/ijms20123088

**Published:** 2019-06-24

**Authors:** Maria A. Boldyreva, Evgeny K. Shevchenko, Yuliya D. Molokotina, Pavel I. Makarevich, Irina B. Beloglazova, Ekaterina S. Zubkova, Konstantin V. Dergilev, Zoya I. Tsokolaeva, Dmitry Penkov, Mu-Nung Hsu, Yu-Chen Hu, Yelena V. Parfyonova

**Affiliations:** 1 National Medical Research Center of Cardiology, Russian Ministry of Health, 121552 Moscow, Russia; mboldyreva@inbox.ru (M.A.B.); yulia.molokotina@gmail.com (Y.D.M.); irene.beloglazova@gmail.com (I.B.B.); cat.zubkova@gmail.com (E.S.Z.); doctorkote@gmail.com (K.V.D.); tsokolaevazoya@mail.ru (Z.I.T.); dpenkov@yahoo.com (D.P.); yeparfyon@mail.ru (Y.V.P.); 2 Faculty of Medicine, Lomonosov Moscow State University, 119991 Moscow, Russia; 3 Institute for Regenerative Medicine, Lomonosov Moscow State University, 119191 Moscow, Russia; pavel.makarevich@gmail.com; 4 Department of Chemical Engineering, National Tsing Hua University, Hsinchu 300, Taiwan; peter5552345@hotmail.com (M.-N.H.); ychu@mx.nthu.edu.tw (Y.-C.H.); 5 Frontier Research Center on Fundamental and Applied Sciences of Matters, National Tsing Hua University, Hsinchu 300, Taiwan

**Keywords:** hepatocyte growth factor, adipose derived stromal cells, cell sheet, hind limb ischemia, skeletal muscle regeneration, angiogenesis, reinnervation

## Abstract

Cell therapy remains a promising approach for the treatment of cardiovascular diseases. In this regard, the contemporary trend is the development of methods to overcome low cell viability and enhance their regenerative potential. In the present study, we evaluated the therapeutic potential of gene-modified adipose-derived stromal cells (ADSC) that overexpress hepatocyte growth factor (HGF) in a mice hind limb ischemia model. Angiogenic and neuroprotective effects were assessed following ADSC transplantation in suspension or in the form of cell sheet. We found superior blood flow restoration, tissue vascularization and innervation, and fibrosis reduction after transplantation of HGF-producing ADSC sheet compared to other groups. We suggest that the observed effects are determined by pleiotropic effects of HGF, along with the multifactorial paracrine action of ADSC which remain viable and functionally active within the engineered cell construct. Thus, we demonstrated the high therapeutic potential of the utilized approach for skeletal muscle recovery after ischemic damage associated with complex tissue degenerative effects.

## 1. Introduction

Decades of experimental and clinical research in the field of peripheral vascular disease have still not led to the full-scale translation of therapeutic angiogenesis techniques into clinical practice. The complexity of ischemic tissue damage and imperfection of the developed methods together have determined the failure to reach sufficient efficacy when applied in humans.

Current pharmacological management of ischemic disorders mostly addresses metabolic risk factors, while surgical revascularization is applied at an advanced stage of disease to prevent critical tissue damage or organ loss, though this is inapplicable for a large cohort of patients due to elder age, obesity and other comorbidities. Thus, an unmet need for stimulation of the regenerative process after ischemic injury exists, and despite our ability to induce angiogenesis and drive tissue protection, we are yet to achieve full recovery after tissue loss. The therapeutic angiogenesis approach, utilizing exogenous angiogenic stimuli application, holds promise for ischemia treatment. The use of recombinant proteins and gene therapy methods failed to reach primary endpoints in clinical trials of therapeutic angiogenesis [1,2]. This is believed to have happened due to a short residence time for recombinant factors, insufficient tissue transduction by genetically engineered constructs and, above all, the limited therapeutic potential of these stimuli introduced as a monotherapy.

Peripheral vascular disease is known to be a complex ischemic tissue pathology—initiated by atherosclerosis, it progresses down to vessel stenosis and occlusive lesions. Circulation failure is accompanied by multiple degenerative processes in ischemic tissue, with not only vascular network disruption and muscle tissue damage, but inflammation and neuropathy, particularly in diabetes mellitus patients [3]. To initiate and support tissue regeneration, a multifactorial impact should be applied to the damaged area. An ideal therapeutic would possess tissue protective, matrix-remodeling, angiogenic, vessel-stabilizing and neuroprotective or even neuroregenerative effects.

Though gene therapy continuously improves transgene delivery and expression along with the use of therapeutic factors in combinations, cell therapy remains the most prospective method for ischemia treatment. Since differentiation potential of transplanted cells is debatable, their paracrine activity is suggested to be the primary determinant of therapeutic impact. Among different stem and progenitor cell types used for experimental therapeutic angiogenesis, the accessible and easily expansible adipose-derived mesenchymal stromal cells (ADSC) are most prospective [4]. These cells secrete a wide variety of angiogenic, arteriogenic and antiapoptotic factors essential for tissue protection and vascularization [5,6,7]. Moreover, there is convincing evidence that ADSC can be incorporated into the vessel wall directly during angiogenesis and act as pericytes [8,9]. We previously showed that ADSC were effective in vascular network development, supporting collateral remodeling and blood flow restoration in the rodent model of limb ischemia [10,11,12]. The effect was greater, as expected, when ADSC, gene-modified by VEGF165-coding viral vector, were used. Importantly, such therapy led not only to capillary density increase but promoted arteriogenesis supporting evidence for vessel stabilizing factors secretion by cells delivered [13]. 

Considering neuropathic conditions in ischemic tissue, particularly in patients with diabetic baseline neuropathy, it is reasonable to strengthen ADSC therapeutic potential by enhancing their neuroprotective action. Among candidates for delivery to mesenchymal stromal cells (MSC), hepatocyte growth factor (HGF) possesses pleiotropic effects on inflammatory response, blood vessel maturation, Schwann cells activation and neuronal protection [14,15,16]. Our own data indicate that HGF expression in myocardium or ischemic skeletal muscle models stimulates neovascularization, suppresses excessive inflammation and reduces tissue necrosis span [17]. In a traumatic nerve injury model, we previously found out that plasmid-based HGF gene therapy accelerated functional and anatomical recovery of the peroneal nerve [18]. 

While the regenerative potential of cell therapy for ischemic disorders was shown in numerous experimental studies, its efficacy in clinical research was underwhelming in many cases. The main limitation is low viability and low retention of delivered cells in target tissue [19]. Thus, strategies to improve survival, tissue incorporation and functional activity of transplanted cells are urgently needed for successful cell therapy. Accumulated data indicate that cell delivery in the form of multilayered cell sheets (CS) bears a significant advantage—mostly due to the fact that cells are transplanted along with extracellular matrix (ECM) formed—that supports their survival and tissue engraftment. In previous studies, we demonstrated that transplantation of ADSC via cell sheets was superior to an equivalent dose of suspended cells in therapeutic potential [11,12].

The present study focuses on the potential to recover vascular and neural trophic in ischemic skeletal muscle after delivery of a multilayered cell sheet comprised of HGF-expressing MSC sheets in a mouse model of limb ischemia.

## 2. Results

### 2.1. Characteristics of Cell Sheets Comprised of HGF-Overexpressing ADSC

**Figure 1:**
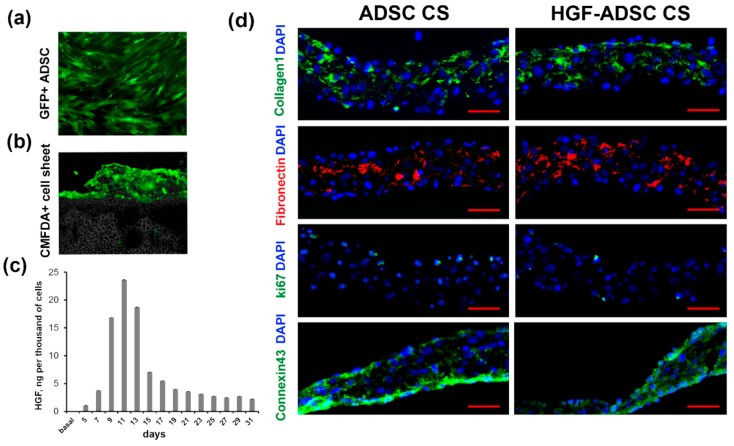
Characteristics of cell sheets comprised of adipose-derived stromal cells (ADSC). (**a**) GFP-expressing ADSC transduced by rAAV-GFP; (**b**) CMFDA-labeled cell sheet covering skeletal muscle; (**c**) hepatocyte growth factor (HGF) production by gene modified ADSC evaluated by ELISA; (**d**) representative images of cell sheet frozen sections stained against collagen (green), fibronectin (red), ki67 (green), connexin 43 (green) and DAPI (blue). Scale bar = 50µm.

Mouse ADSC were transduced with chimeric adeno-associated viral vector serotype (AAV-DJ) encoding mHGF or green fluorescent protein (GFP). According to FACS analysis, the efficacy of transduction using GFP-encoding virus (Figure 1a) reached 90%. The enzyme-linked immunosorbent assay (ELISA) showed an increased production of HGF by modified ADSC (HGF-ADSC). The maximum level of HGF was 23.6 ng/10^3^ cells/48 h on day 11 after infection (Figure 1c), which was 100-fold higher than the basal protein production by non-transduced cells (0.015 ng/10^3^ cells/48 h). By day 31, the level of HGF decreased to 2.2/10^3^ cells/48 h. Cell sheets comprised of HGF-overexpressing ADSC (HGF-ADSC CS) were generated as described in Methods section. On day 5, following viral transduction, no difference was found in the level of HGF production between HGF-ADSC (1.09 ng/10^3^ cells/48 h) and HGF-ADSC CS (1.10 ng/10^3^ cells/48 h). The limited time of cell sheet assembly (<2–3 days) prevented transgene production analysis at later timepoints; if cultured longer, CS underwent spontaneous detachment and contraction. Immunofluorescent staining against Connexin 43 showed the formation of gap junctions between cells in CS. Analysis for collagen and fibronectin (Figure 1d) showed efficient extracellular matrix production within CS, comprised both of ADSC or HGF-ADSC. Sporadic ki-67-positive cells were detected in CS, indicating a proliferation predominantly along the edges.

### 2.2. Stimulating Effect of Conditioned Medium Collected from HGF-Producing ADSC on Neurite Outgrowth and Glial Cells Migration In Vitro

**Figure 2:**
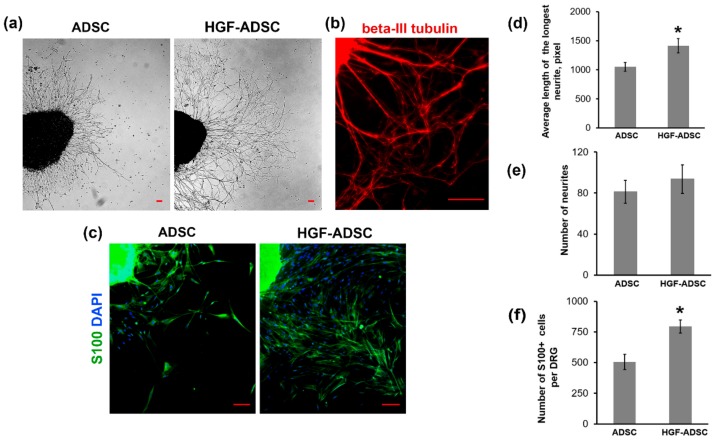
Effects of conditioned medium from HGF-producing or unmodified ADSC on neurites length and number of glial cells in dorsal root ganglion (DRG) explant model. (**a**) Phase-contrast DRG explant images; (**b**) immunofluorescence staining of DRG against beta-III tubulin; (**c**) representative immunofluorescence images of DRG explants stained against S100 (green) and DAPI (blue); (**d**–**f**) quantification of the average length of the longest neurite (**d**), number of neurites (**e**) and number of S100+ cells migrated from DRG explants (**f**). Data are presented as mean ± standard deviation (* *p* < 0.0001, Student’s *t*-test). Scale bar = 100µm.

To estimate the neuroprotective potential of HGF-ADSC the dorsal root ganglion (DRG) explant model was used. The DRG was incubated in conditioned medium obtained from ADSC or HGF-ADSC, stained for neuronal marker β-III tubulin (Figure 2a,b) and subjected to analysis. We found that neurite outgrowth, represented as the average length of the longest neurite, was 1.4-fold higher in the HGF-ADSC group (1414 ± 122, pix) than in the ADSC group (1051 ± 76, pix) (* *p* = 0.0002; Figure 2a,d). We detected a slight increase in the number of neurites in the HGF-ADSC group (93.75 ± 14) compared to ADSC (81.25 ± 11), but the difference did not reach significance (*p* = 0.06; Figure 2e). Immunofluorescent staining against glial marker-S100 was performed to demonstrate the stimulating effect of conditioned medium from HGF-ADSC on glial cell migration. The number of glial cells that migrated from DRG explant was 1.6-fold higher in HGF-ADSC group compared to unmodified cells (795 ± 54 vs. 506 ± 63, respectively; * *p* < 0, 00001) (Figure 2c,f).

### 2.3. Blood Flow Recovery Following HGF-Producing ADSC CS Transplantation into Ischemic Limb 

**Figure 3:**
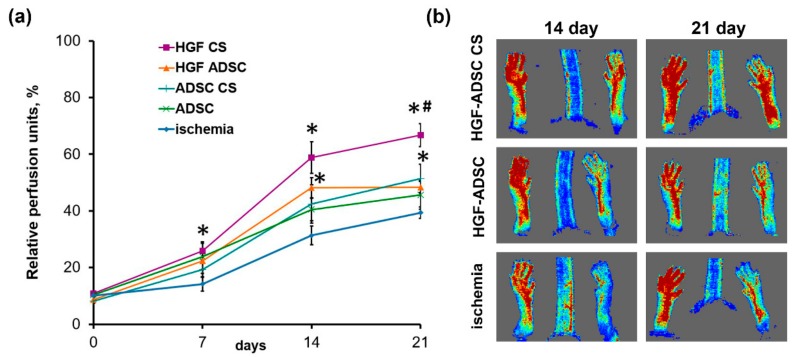
Blood flow recovery in ischemic mouse after transplantation of HGF-producing ADSC sheet. (**a**) Chart reflects dynamics of limb perfusion in ischemia group (*n* = 9) or treated animals that received HGF-ADSC CS (*n* = 10), ADSC CS (*n* = 10), suspended HGF-ADSC (*n* = 9) or ADSC (*n* = 8); *- vs. untreated control, #- vs. HGF ADSC group. (**b**) Representative laser-doppler images of subcutaneous blood flow at day 14 and 21 after ischemia induction and constructs/cells transplantation. HGF = hepatocyte growth factor, CS = cell sheet, ADSC = adipose-derived stromal cells.

In order to evaluate the therapeutic effect of ADSC-derived cell sheets on tissue recovery after ischemic injury, we utilized the murine hind limb ischemia model. Treated groups were (1) ADSC or (2) HGF-ADSC suspension, and (3) ADSC or (4) HGF-ADSC derived CS. The CS were applicated on the area of femoral artery ligation, while cell suspensions were injected intramuscularly. Histological analysis of tissue sections (see Methods section) obtained at day 14 following surgery and transplantation confirmed persistence and viability of transplanted labeled cell constructs (Figure 1b). Moreover, cells labeled by CMFDA prior to CS formation were found subjacent to the construct (within skeletal muscle), indicating ADSC migration from CS. We found that both ADSC suspensions and CS promoted restoration of hind limb perfusion demonstrated by laser doppler imaging. At day 7, after transplantation, statistical significance was found only between HGF-ADSC CS and untreated groups (25.85% ± 3.21% vs. 14.19% ± 2.52%, respectively; *p* = 0.012). By day 14, all treated groups showed blood flow recovery (Figure 3a,b) superior to spontaneous reperfusion in the control untreated group. Though no significant difference was observed between HGF-ADSC CS and HGF-ADSC groups at day 14, the use of HGF-ADSC CS was more effective compared to cell suspensions (statistically significant). Nonetheless, at day 21, the blood reperfusion in animals treated with HGF-ADSC CS reached 67% (1.7-fold higher than in the untreated group (40%, * *p* = 0.004)), and became significantly higher than the HGF-ADSC (48.40% ± 1.89%, # *p* = 0.020) group.

### 2.4. Increased Vascularization of Ischemic Skeletal Muscle after HGF-ADSC CS Transplantation

**Figure 4:**
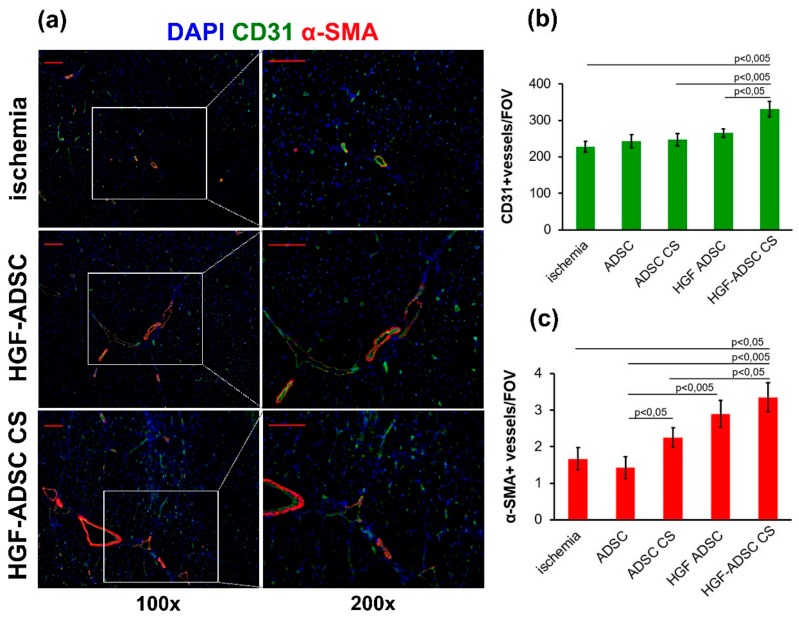
Blood vessel density in ischemic muscle at day 14 after ischemia induction and cell sheets/cells transplantation. (**a**) Representative images of *m. tibialis anterior* sections from ischemia, HGF-ADSC and HGF-ADSC CS groups stained against murine CD31, α-SMA and DAPI (100× and 200× magnification); (**b**,**c**) graphical presentation of blood vessel density analysis with average group values per FOV. Data are presented as mean ± SEM (Mann–Whitney U-test). Scale bar = 50μm. HGF = hepatocyte growth factor, CS = cell sheet, ADSC = adipose-derived stromal cells. α-SMA = α-smooth muscle actin, DAPI = 4′,6-diamidino-2-phenylindole, FOV = field of view.

Analysis of vascular density in ischemic *m. tibialis anterior* was obtained at day 14 after ischemia induction and cell or CS transplantation and after being stained against endothelial and smooth muscle cells specific markers (Figure 4). The maximum capillary density was found in the HGF-ADSC CS group that was higher than in other experimental groups and almost 1.5-fold higher compared to the untreated group. Although the trend to increase was clear, the capillary number did not reach statistical significance between ADSC, ADSC CS, HGF-ADSC, and untreated groups. Interestingly, the α-SMA-positive vessel (indicative of arteriogenesis) count showed that transplantation of both HGF-ADCS and HGF-ADSC CS increased their density compared to the control (Figure 4c). The number of larger vessels in the ADSC and ADSC CS groups did not increase compared to untreated animals. Still, application of ADSC CS was more effective than cell transplantation as a suspension.

### 2.5. Neuroprotection in Ischemic Skeletal Muscle Following HGF-Producing CS Transplantation Overexpression

**Figure 5:**
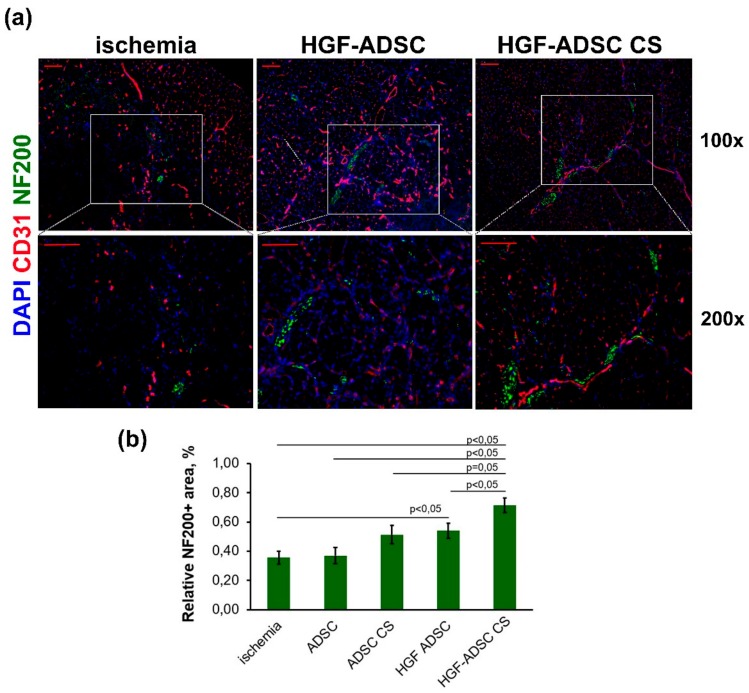
Neuronal innervation in ischemic muscle at day 14 after ischemia induction and cell sheets/cells transplantation. (**a**) Representative images of *m. tibialis anterior* sections from ischemia, ADSC, ADSC CS, HGF-ADSC and HGF-ADSC CS groups stained against murine NF200, CD31 and DAPI (100× and 200× magnification); (**b**) graphical presentation of neuronal innervation assessment using relative area of NF200+ structures per FOV. Data are presented as mean ± SEM (Mann–Whitney U-test). Scale bar = 50μm. HGF = hepatocyte growth factor, CS = cell sheet, ADSC = adipose-derived stromal cells. NF200 = Neurofilament 200, DAPI = 4′,6-diamidino-2-phenylindole, FOV = field of view.

Considering the results from the DRG model, which demonstrated nerve growth stimulation by HGF-producing CS, we next examined muscle tissue samples for neuronal innervation. To that end, immunofluorescent staining for the NF200 axon marker was performed at day 14 after surgery and transplantation (Figure 5). Estimating the relative NF200+ area, we found no difference between the untreated group (0.36% ± 0.04%) and animals treated with unmodified ADSC either in suspension (0.37% ± 0.05%, *p* = 0.89) or in CS (0.51% ± 0.06%, *p* = 0.09). By contrast, HGF-producing cells promoted a significant increase in the NF200+ area, reaching 0.54% ± 0.05% and 0.71% ± 0.05% for HGF-ADSC and HGF-ADSC CS, respectively (*p* = 0.028 and *p* = 0.001 vs. control group, respectively) (Figure 5b). 

### 2.6. Reduction of Necrosis and Anti-Fibrotic Effect after ADSC Transplantation to Mice with Limb Ischemia

**Figure 6:**
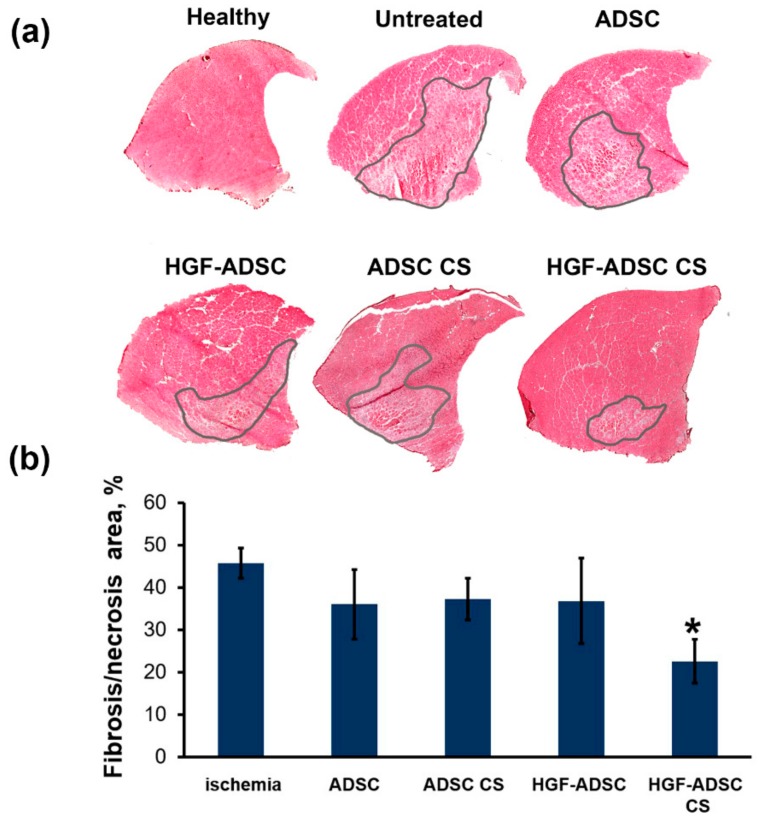
Morphometric analysis of necrosis/fibrosis in ischemic muscle at day 14 after ischemia induction and cell sheets/cells transplantation. (**a**) Whole-section images of hematoxylin/eosin stained *m. tibialis anterior*. Necrotic/fibrotic tissue is marked by a line; (**b**) graphical presentation of necrotic/fibrotic tissue span. Data are presented as mean ± SEM (Mann–Whitney U-test; * *p* = 0.025 vs. untreated ischemic group). HGF = hepatocyte growth factor, CS = cell sheet, ADSC = adipose-derived stromal cells.

The necrotic tissue-span in ischemic muscle, defined by morphology changes, inflammatory infiltration and fibrosis, was visualized by hematoxylin-eosin staining and correlated with angiogenic and neuroprotective effects of HGF-producing cell sheets observed in respective experiments. All treated groups showed a reduction of necrotic tissue area compared to control (Figure 6). The most significant 2-fold reduction was shown for HGF-ADSC CS group (22.6% ± 5.2% vs. 45.7% ± 3.5% in control; * *p* <0.05). No significant difference was found between ADSC-treated animals (36.0% ± 8.2% for ADSC, 37.3% ± 4.9% for ADSC CS, 36.8% ± 10.1% for HGF ADSC, 22.6% ± 5.2% for HGF CS).

## 3. Discussion

In the present study, we investigated a novel approach for therapeutic angiogenesis by applying HGF-ADSC sheet technology to improve functional and morphological recovery in the ischemic limb. We found a superior therapeutic efficacy of HGF-ADSC sheet compared both to HGF-ADSC suspension or untransduced ADSC sheet in blood flow recovery, vascularization and innervation of ischemic skeletal muscle. 

ADSC are considered the most promising cell source for cell therapy because of their regenerative capacity due to secretion of pro-survival soluble factors, production of extracellular matrix proteins, and their immunomodulating properties [20,21]. However, results of numerous cell therapy clinical trials, including those with ADSC, did not meet initial expectations [22,23]. In those studies, conventionally used direct injections of cell suspensions showed limited efficacy, putatively due to low cell viability as a result of cell-to-cell interaction loss, compression damage during administration by injection, and the unfavorable microenvironment in ischemic and inflammatory tissue. To overcome this issue, an approach known as “cell sheet engineering” can be used; it relies on generation of scaffold-free tissue-like constructs consisting of cells and extracellular matrix as well as growth factors produced by the cells. Recently, this approach to cell delivery preserving viability and supporting cells’ regenerative functions, has been applied in experimental and clinical studies in regenerative medicine [24,25,26,27,28]. 

In this study, we simultaneously used two approaches to augment the regenerative capacity of ADSC. First, the cells were modified with adeno-associated virus encoding HGF gene to enrich ADSC secretome and enhance therapeutic potential. Second, gene-modified ADSC were transplanted to ischemic limb as CS to enhance their survival, and preserve their viability and regenerative functions. Utilization of CS technology for ischemic skeletal muscle and myocardium regeneration was effective in a number of studies, including our own [11]. Specifically, in hind limb ischemia smooth muscle, cell sheets transplanted over the femoral artery-resected area induced blood flow recovery and capillary enrichment [29]. 

Regeneration as a process requires not only activation of stem cells by specific signals after damage, but also a complex of coordinated events that leads to formation of normal tissue on the site of injury. From this point of view, the delivery of HGF-expressing cells seems to be a pleiotropic treatment approach impacting:

(1) Angiogenesis and vascularization of tissue
(2) Neural terminals and innervation
(3) Immune response and control of excessive inflammation
(4) Reduction of fibrosis and necrosis span

Thus, the suggested approach may go beyond a feasible tool to deliver HGF via genetically-modified CS. Considering therapeutic angiogenesis, HGF demonstrates a unique capability to stimulate vessel growth without causing inflammation [17,30]. Recent studies of skeletal muscle renewal and regeneration suggest a critical role of HGF/c-met axis in the activation of satellite cells in skeletal muscle niche. For a decade, HGF was considered the only known growth factor to recruit satellite cells rendering full recovery of skeletal muscle in mammals [31]. Later, the role of TGF (transforming growth factor), IGF (insulin-like growth factor) and other growth factors was established, but HGF is known to prime the quiescent satellite cells for the “alert” stage under weak injury. However, if injury persists and signals cross the threshold, alerted satellite cells will be quickly activated, and muscle will be efficiently repaired [32]. A significant angiogenic effect of HGF-producing cells was confirmed in myocardial ischemia models [33,34] and in a rat model of hind limb ischemia [35]. Moreover, HGF-expressing mesenchymal stromal cells were more efficient in a rat transient middle cerebral artery occlusion model compared to unmodified cells [36]. 

The present study shows that transplantation of HGF-ADSC as cell sheet results in more effective blood flow recovery, vascularization and innervation of ischemic hind-limb compared to HGF-ADSC suspension or ADSC sheets. We suggest that the observed effects are associated with higher survival rate of ADSC within the cell sheet compared to suspended cells and, thus, more prolonged paracrine activity. HGF production by ADSC, aside from supporting viability of transplanted cells through autocrine manner, promotes anti-inflammatory and anti-fibrotic effects. The observed effects are further strengthened by other growth factors secreted by ADSC, particularly vascular endothelial growth factor (VEGF). Our previous data demonstrate the cooperative effect of VEGF165 and HGF overexpression on induction of angiogenesis and fibrosis reduction evaluated in mouse ischemic limb [37] and myocardial infarction in rats [17]. HGF is well-known for its antifibrotic effect, which is of crucial importance as far as post-traumatic fibrosis virtually excludes replacement by contractile tissue. This “rule of thumb” applies to most human tissues where scar plays a role of “roadblock” for any functional element (parenchyme) to appear. Antifibrotic effects of HGF rise from its ability to reduce TGF-b production by ECM-producing myofibroblasts and counteract effects of TGF by negative modulation of smad 2/3 pathway [38]. Finally, HGF suppresses cell sensitivity to TGF-b by reduction of TGF-b receptor [39] and increase of decorin—a natural counter-partner of TGF-b activation [40]. The role of HGF in suppression of fibrous degeneration after ischemic damage was shown previously [41] and was attributed to the reduction of collagen types I and III synthesis by cardiac fibroblasts expressing c-met receptor [42].

Alongside vascular density improvement, we found that HGF-producing ADSC sheet transplantation led to better innervation of ischemic muscle. This point is significant, as the revascularization process requires appropriate arterial innervation to efficiently restore tissue function. Arteries are innervated by postganglionic sympathetic nerve fibers that promote post-ischemic revascularization processes through several distinct mechanisms [43]. First, catecholamines released from nerve terminals contribute to collateral growth and angiogenesis in mice hindlimb ischemia [44]. Moreover, the sympathetic nervous system is known to modulate hematopoietic stem/progenitor cells mobilization and homing to ischemic tissues through endothelial nitric oxide synthase activation [45] and regulation of CXCL12 chemokine [46]. In addition, communication with immune cells by sympathetic nerves modulates post-ischemic inflammation [43,44]. We observed an increase in nerve endings density 14 days after skeletal muscle ischemia modelling and cell sheet application. This suggests an accelerated restoration of muscle innervation, which might occur through the promotion of both regrowth and maintenance of damaged axons by HGF and other neuroprotective and neurotrophic factors of ADSC secretome. The survival and functioning of neurons, as well as regrowth of axons, depends on trophic support by glial cells and continuous supply of growth factors by skeletal muscles [47]. It is known that Schwann cells and neurons are vulnerable to the ischemia consequences [48]. ADSC secretome, enriched by HGF, possesses strong neurotrophic and neuroprotective activity [49] and might contribute to the reinnervation of ischemic muscle. We found that conditioned medium collected from HGF-ADSC stimulated neurite outgrowth from dorsal root ganglia explant, promoted migration and proliferation of glial cells. We suggest that enhanced revascularization by HGF-ADSC sheets and HGF-specific action promote re-innervation of ischemic muscles, which, in turn, further accelerates angiogenesis, thus providing cooperative regenerative impact. One could speculate that an increased rate of axon growth may allow nerves to achieve targets faster during regeneration, leading to enhanced functional recovery of impaired tissue. HGF expressed by ADSC may affect axonal regrowth directly by activation of c-met or through cooperative action with glial cell-derived neurotrophic factor (GDNF) expressed by the proliferating vasculature [50]. We previously showed that the combined action of GDNF and HGF stimulated axonal growth via the cooperative induction of Erk1/2 phosphorylation and downstream signaling activation [51]. Apart from paracrine activity, the HGF-producing cell sheet can stimulate Schwann cells to proliferate and migrate, thus providing trophic support to regenerating axons. 

## 4. Materials and Methods 

### 4.1. Cell Cultures

Mouse ADSC (mADSC) were isolated from subcutaneous adipose tissue of male C57/Bl6 mice as previously described [11]. Isolated cells were cultured in 4.5 g/L D-glucose DMEM containing 10% fetal bovine serum (FBS) (Gibco, Waltham, MA, USA) and 1% antibiotic/antimycotic solution. A human embryonic kidney (HEK-293T) cell line was purchased from ATCC and cultured in Dulbecco’s modified Eagle’s medium (DMEM) (Paneco, Moscow, Russia) containing 10% FBS (Gibco, USA) and 1% antibiotic/antimycotic solution. Cell cultures were maintained in a humidified chamber at 37 °C and 5% CO_2_. For all experimental procedures, including CS generation and preliminary testing, early passage cells were used (P3–P4).

### 4.2. DNA Constructs, Viral Vectors and Cell Transduction

The mouse HGF coding sequence (NM_001289458.1) flanked with BamH1 and Sal1 restriction sites (Kozak consensus sequence was added upstream ATG codon as well) was synthesized and cloned into pUC57 plasmid by Genescript (Genescript, Piscataway, NJ, USA). Subsequently, the gene was subcloned to pAAV-MCS using BamH1, Sal1 enzymes to generate pAAV-mHGF. To produce recombinant AAV (rAAV) coding, mouse HGF HEK293T cells were transfected according to AAV Helper-Free System (Stratagene, San Diego, CA, USA) protocol. Small-scale vector preparations were made in 100 mm dishes by cotransfection of HEK293T cells with plasmids pAAV-DJ (Cell Biolabs, San Diego, CA, USA), pHelper (Stratagene, San Diego, CA, USA) and pAAV-mHGF.

HEK293T cells that reached 80% confluency were transfected using calcium-phosphate co-precipitation method with 10 μg of each plasmid per 100-mm dish. Transfected cultures were maintained for 48–54 h at 37 °C in DMEM supplemented with 10% FBS. Thereafter, cells were detached and collected by centrifugation at 200 × *g* for 10 min. The cell pellet was resuspended with 1 mL of PBS (per culture dish) and was subjected to four freeze–thaw cycles. Cell lysate was then incubated with 50 U/mL of Benzonase (Merck, Darmstadt, Germany) at 37 °C for 30 min, followed by cell debris removal by centrifugation (5000× *g* for 25 min). The supernatant (viral stock) was aliquoted and stored at −70 °C until use. For ADSC transduction, the cells were grown in a 100 mm culture dish until 70% confluency was reached. The media was then replaced with 2 mL of DMEM + 3 mL of viral stock. The dish was agitated by tapping to ensure even cell distribution, and incubated at 37 °C, 5% CO_2_ (with tapping every 30 min). Three hours after infection, 5 mL of DMEM, containing 20% fetal bovine serum (FBS) (Gibco, Waltham, MA, USA) and 2% antibiotic/antimycotic solution, were added. Infected cells were cultured for 48 h prior to experiments.

### 4.3. Cell Sheets

For cell sheet formation, mADSC were seeded at 1 × 10^6^/well in a 12-well culture plate (Corning, Corning, NY, USA) and incubated at 37 °C, 5% CO_2_ for 48 h. For detachment the CSs were washed and incubated in Versene solution (at 37 °C 5% CO_2_) until they self-detached in 2–5 min. After that, CSs were transferred to DMEM. To monitor HGF production in cell and CS culture medium we used enzyme-linked immunosorbent assay HGF (#100686, Abcam, Cambridge, UK). For cell sheet labeling, CellTracker™ Green CMFDA (5-chloromethylfluorescein diacetate) dye (1:2000 dilution; Thermo Scientific, Waltham, MA, USA) was added to CS culture medium 1 h prior to detachment and transplantation. Suspended mADSC were labeled by CellTracker PKH26 Sigma-Aldrich, Milwaukee, WI, USA) according to the manufacturer’s protocol in PBS. Afterwards, CS or suspended mADSC were washed and observed under a fluorescent microscope prior to transplantation to ensure dye incorporation. Stained CS were visualized on fresh, unfixed biceps femoris section, isolated on day 14 from the transplantation zone.

### 4.4. Animals

The 9–10-week-old male C57/Bl6 mice were purchased from Laboratory Animals Nursery “Andreevka”, National Center of Biomedical Technologies, Russia. Following an acclimation period, animals received standard food and water ration according to in-house husbandry rules. All animals were narcotized by intraperitoneal injection of avertin (300 μL of 2.5% solution) before surgery. Euthanasia was conducted under isoflurane narcosis by secondary cervical dislocation. Surgical manipulations and euthanasia procedures were developed in compliance with national and European Union directives and were approved by the Institutional Ethics Board for Animal Care (National Medical Research Center of Cardiology; permit #34 (23.01.2017).

### 4.5. Dorsal Root Ganglion Explants

The experiments were performed on neonatal C57/Bl6 mice (*n* = 8). Dorsal root ganglia (DRG) were isolated under aseptic conditions using microsurgical sterile instruments. The vertebral column of the decapitated animal was released from the surrounding tissues on the dorsal side, after which the spine was opened, the spinal cord was removed, and spinal ganglia were extracted, cutting off the nerve branches as much as possible. Explants were plated on 35 mm cell dishes, which were pretreated by poly-D-lysine solution (0,1 mg/mL; Sigma-Aldrich, Milwaukee, WI, USA) and cultivated in DMEM (Gibco, Waltham, MA, USA), 4,5 g/l glucose, supplemented by 10% fetal bovine serum (HyClone, Logan, UT, USA) and 1% solution of PenStrep (Gibco, Waltham, MA, USA) (37 °C and 5% CO_2_), 4 h prior to attaching explants to the substrate. Afterwards, the cultured medium was replaced by conditioned medium collected from HGF-modified ADSC. The final concentration of HGF was 135 ng/mL. DRGs incubated in medium collected from unmodified ADSC were used as a control. Explants were cultivated at 37 °C and 5% CO_2_ for 48 h. The morphological assay of DRG explants was evaluated by Zeiss Axio Observer A1 microscope (Zeiss, Oberkochen, Germany). For the immunofluorescence assay, DRG explants were fixed in 4% formaldehyde solution on PBS (10 min, room temperature), washed by PBS and incubated in 0.1% Triton-X100 solution. After that, explants were washed and blocked in 10% serum of secondary antibody donor solution. Fixed tissues were stained by primary rabbit monoclonal antibody against β-III tubulin (1:100; #ab68193, Abcam, Cambridge, MA, USA) and S100B (1:100, #B80198, Sigma-Aldrich, Milwaukee, WI, USA) for 12 h at 4 °C and by Alexa Fluor^®^ 594-conjugated secondary antibody (#11037, Molecular Probes, Carlsbad, CA, USA), or by Alexa Fluor^®^ 488-conjugated secondary antibody (#11034, Molecular Probes, Carlsbad, CA, USA), respectively (1:800; 1 h, room temperature, darkness). Neurite outgrowth was evaluated by two parameters: The average length of the longest neurite of DRG and number of neurites. For length of the longest neurite measurement, we determined 10 to 15 neurites and calculated the average length of the 5 longest neurites for each DRG (ImageJ Software, NCI, Bethesda, MD, USA) [52,53]. The number of neurites was evaluated using the method of Zhang et al. [54]. DRG explants stained against S100 were used to quantify the number of S100+ glial cells per FOV. The statistically significant difference between groups was determined using a Student’s *t*-test.

### 4.6. Mouse Hind Limb Ischemia Model and Cell Transplantation

C57/Bl6 male mice were narcotized by intraperitoneal injection of 2.5% avertin solution. All surgical manipulations were carried out in aseptic conditions under a binocular microscope Leika M620 TTS (Leika Microsystems, Wetzlar, Germany). Unilateral induction of hind limb ischemia was carried out as previously described [55]. Briefly, skin was incised along the midline of the left hind limb and the femoral artery, with its branches, was ligated between its proximal part and popliteal bifurcation. The blood vessel was excised between the upper and lower ligatures with the sciatic nerve kept intact. After that, the CS was transplanted in a drop of PBS to cover the site of excised blood vessels and dried by a cotton ball. Animals that received CS transplantation formed ADSC CS or HGF-ADSC CS groups (*n* = 10/group). In the untreated control ischemia group (*n* = 9), the wound was rinsed with PBS and dried. After CS adhesion (for 1–2 min), the skin was closed with 5–0 silk sutures and animals were placed in a chamber on a heated pad until full recovery. Additionally, mice that received ADSC suspension injection formed ADSC (*n* = 8) and HGF-ADSC (*n* = 9) groups, respectively. A total of 1.0 × 10^6^ cells ADSC were diluted in 150 μL HBSS. Cells were delivered in three equal injections (50 μL each) to the anterior tibia muscle, the femoral biceps muscle and the femoral quadriceps muscle. After surgery, all animals received a 1.5 mL bolus of warm sterile saline subcutaneously to compensate for blood loss.

### 4.7. Laser Doppler Perfusion Measurement

Subcutaneous blood flow was assessed using Laser Doppler Imaging System (Moor Instruments Ltd, Millwey, UK) as previously described [55]. Blood perfusion was measured immediately and then at day 7, 14 and 21 after surgery. Animals were narcotized by avertine intraperitoneal injection. Perfusion measurements (*n* = 3–4) on the plantar surface of the animal’s feet were made and data variability was analyzed using Moor Image Review software. Readings were taken until three subsequent runs with minimal (<10%) deviation were obtained. To account for variability among measurements, limbs and expressed as relative perfusion (%).

### 4.8. Histological Analysis

At day 14 after surgery and CS transplantation, animals (*n* = 4–5 for each group) were sacrificed by lethal isoflurane inhalation. After skin dissection, the femoral quadriceps and tibia anterior muscles were harvested and frozen in TissueTek medium (Sakura Finetek, Leiden, the Netherlands). Parallel frozen sections (7 μm) were prepared (using Microm HM 505E, MICROM International GmbH, Walldorf, Germany) on glass slides and stored at −70 °C until staining. For Immunofluorescent analysis, *m. tibialis anterior* sections were fixed in ice-cold acetone for 20 min, airdried and washed in PBS (5 min). All antibodies were diluted in blocking solution consisting of 1% BSA in PBS. After washing, slides were blocked by 10% normal donkey serum (30 min), washed and incubated overnight with primary antibodies (rabbit anti-NF200 antibody, #4142, Sigma-Aldrich, Milwaukee, WI, USA; rat anti mouse CD31, #550274, BD Biosciences Pharmingen, San Diego, CA, USA; rabbit anti α-SMA antibodies, #5694550274, Abcam, Cambridge, MA, USA). After that, the sections were stained with Alexa Fluor^®^ 488-conjugated secondary antibody (#A21206, Thermo Scientific, Waltham, MA, USA) or with Alexa Fluor^®^ 594-conjugated secondary antibody (#A21209, Thermo Scientific, Waltham, MA, USA) (1:800) for 1 h; all slides were counterstained with DAPI (4′,6-diamidino-2-phenylindole) dye (Sigma-Aldrich, Milwaukee, WI, USA). Staining was visualized on a fluorescent microscope Zeiss Axio Observer A1 (Zeiss, Oberkochen, Germany). Obtained images were analyzed using ImageJ freeware. Microphotographs were taken under 200 × magnification. For neural innervation quantitative assay, the relative NF200+ area per FOV was calculated. Capillary CD31+ structures and α-SMA+ blood vessels (from 30 μm in diameter) were counted per FOV (ImageJ, NCI, Bethesda, MDNIH, USA) and used to obtain mean values for section. To calculate, the 2–3 FOVs from 5–6 sections were analyzed for each sample to determine an average number per FOV for each animal and for the whole group. Routine hematoxylin/eosin staining was used for necrotic tissue count. Stained muscles were photographed as described above, and necrotic/viable tissue ratio was calculated using the color threshold function in ImageJ freeware. Obtained data was used for subsequent statistical analysis after being normalized to section area.

### 4.9. Statistical Analysis

Data were expressed as mean ± standard error of the mean (SEM) for each group. Statsoft “Statistica 8.0” was used for analysis of obtained data. Statistically significant differences between the two groups were determined by Mann–Whitney U-test depending on sample distribution profile. Multiple groups were compared using ANOVA with Bonferroni correction for level of significance where required; *p*-values less than 0.05 were considered indicative of significance.

## 5. Conclusions

**Figure 7:**
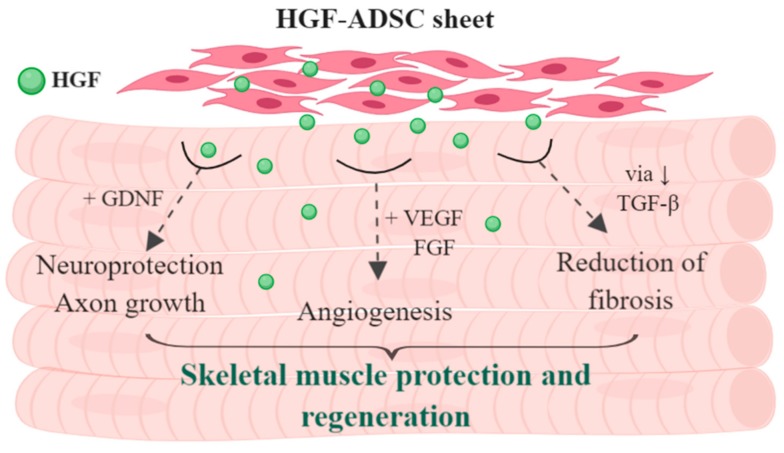
Schematic presentation of supposed pleiotropic effect of HGF-producing ADSC sheet on skeletal muscle regeneration.

In conclusion, the present study demonstrated that HGF-ADSC sheet transplantation into ischemic hind limb of mice provided strong pleiotropic effects (Figure 7), contributing to more effective morphological and functional recovery. This therapeutic strategy that could be applied during vascular surgery holds great promise for the treatment of ischemic peripheral vascular disease, particularly in patients with diabetes mellitus complicated by neuropathies.
